# 

**Published:** 1889-06-22

**Authors:** 


### NOTICE TO CORRESPONDENTS.

All MS., Letters, Books for Review, and other matters intended for the Editor, should be addressed The Editor, The Lodge, Porchester Square, London, W.

### Notice to Advertisers and Subscribers.

All Advertisements, Orders for Copies of The Hospital, and Business Communications should be addressed to The Publisher, *not to the Editor* The Hospital, 139-140, Salisbury-court, Fleet-street, E.C.

Vol. V., now ready, 4s., post free. Cases for binding, may be had of the Publisher, at 1s. 6d. each.

### To Residents, Secretaries, and Matrons.

The Editor will be glad to have the Regulations and each Report as issued by Asylums, Hospitals, Convalescent and Nursing Institutions, as well as those of other Charities, and an early copy in duplicate of all Appeals and Festival Notices, etc., addressed to *The Editor, The Lodge, Porchester-square, London, W.* Any superintendent, secretary, matron, or other chief official who regularly sends such information may be registered, on application to the Publisher, to receive free of cost a cover for binding The Hospital in half-yearly volumes.

### Amusements and Relaxation.

### Word Competition.

Commenced April 6th, Ends June 29th.

Three Prizes of 15s., 10s., 5s., will be given for the largest number of words derived from the words set for dissection.

Proper names, abbreviations, foreign words, words of less than four letters, and repetitions are barred; plurals, and past and present participles of verbs, are allowed. Nuttall's *Standard* dictionary only to be used.

N.B.—Word dissections must be sent in WEEKLY, arranged alphabetically, with correct total affixed.

The word for dissection for this, the *Twelfth* week of the quarter, being

"ORCHARDSON."

### Results of Tenth Week.

| Names. | June 13th. | Totals. | Names. | June 13th. | Totals. |
|---|---|---|---|---|---|
| Patience | 16 | 651 | N'importe Qui | 14 | 520 |
| Nurse Duty | 17 | 635 | Pollie | 15 | 487 |
| Lightowlers | 14 | 631 | W. G. R. | — | 467 |
| Scrooge | — | 541 | Robe | — | 150 |
| Reldas | — | 250 | Beryl | — | 391 |
| Tinie | 16 | 651 | Nesta | — | 124 |
| K. G. | 16 | 605 | Daffodil | — | 89 |
| Broxbourne | 16 | 623 | A Schoolgirl | 10 | 212 |
| Lauriston | 14 | 623 | Montague | — | 79 |
| Spes | 16 | 605 | Sheeny | — | 111 |
| Gretta | — | 224 | Alice R. | — | 63 |
| Francesca | — | 476 | Belfast | — | 163 |
| Heatherbell | — | 185 | Gentian | — | 209 |
| Sycamore | — | 201 | Hope | — | 39 |
| Arctus | — | 499 | Louie | — | 139 |
| Amicus | 15 | 607 | Hamilton | — | 121 |
| Scratcher | — | 197 | E. M. | — | 120 |
| Jemima | — | 138 | F. J. D. | — | 112 |
| Concours | — | 427 | Ladyr | — | 102 |
| C. J. S. | — | 134 | Surgical Nurse | — | 128 |
| See-Saw | 15 | 565 | L. Pearce | — | 32 |
| Rosemont | - | 173 | Contentio | — | 98 |
| Jenny Wren | 15 | 387 | M. W. | 17 | 74 |
| Eric | — | 496 | Esperance | 17 | 75 |
| Tiny | 17 | 561 | H. B. | — | 10 |

### Notice to all Correspondents.

N.B.—All letters referring to this page which do not arrive at 139 and 140, Salisbury-court, London, E.C., by the *first post on Thursdays*, and are not addressed PRIZE EDITOR, will in future be disqualified and disregarded

Competitors can enter for *all* quarterly competitions, but no competitor may take more than *two quarterly prizes* during the year.

### Scraps and Gleanings.

Rabies is on the increase in London.

The new Cottage Hospital at Bingley is to cost £1,300.

Hospital Saturday at Sleaford resulted in the collection of £130.

Cases of sunstroke occur daily at Berlin, owing to the excessive heat.

Dr. Quain has been appointed one of Her Majesty's Physicians Extraordinary.

The Mayor of St. Helens gave a supper lately to the collectors of the local penny-a-week fund.

Mr. J. D. Davies has been elected honorary junior medical officer o the Isle of Wight Infirmary.

The patients in one of the wards of Belfast Royal Hospital lately su scribed 30s. to buy a clock for the ward.

Mauldeth Hospital for Incurables lately held a sale of patients' work, at which Archdeacon Anson was present.

The first annual report of Leicester Homœopathic Dispensary tells of 2,276 applications for advice and 914 home visits paid.

The friendly societies at Watford have decided to hold a hospita demonstration this summer, similar to that held last year.

Messrs. J. S. Morgan and Co. will receive and forward contributions for the relief of the sufferers from the Johnstown disaster.

Mr. Fane de Salis took the chair at the fifth annual meeting of the Harlington and Cranford Cottage Hospital. A good report was read.

A late patient at the Royal National Hospital, Ventnor, has written to the local papers to express his gratitude for the kindness he received.

The Whitley Convalescent Home has a balance in hand of over a thousand pounds. During the last twelve months 1,377 cases were treated.

An ambulance is to be provided for Yarmouth Hospital as a fitting memorial of the late Dr. Clarke's labours in connection with that institution.

A medical man was lately offered £1,000 by the widow of a late patient, but he very generously bade her give the sum to the local hospital.

The Mayor of Bath has presided over a meeting to consider how best the town can be decorated for Prince Albert's visit in July to open the Bath Hospital.

A woman named Mary Ann Worley, residing in Southsea, was lately attacked and severely bitten by a mad cat. The animal was subsequently destroyed.

During the month of May no fewer than forty-three suicides were committed in Vienna, a total which for that month has not been reached for many years past.

The number of physicians and surgeons in the German Empire on April 1st, 1887, was 15,824, including medical officers in the army and navy, who numbered 1,335.

The Countess de Noailles, who resides at Holywell Lodge, near Eastbourne, has just manifested her sympathy with the anti-vaccination movement by subscribing £500 to the general funds of the association.

The Banbury Board of Guardians lately passed a resolution stopping all vaccination prosecutions, on the ground that the casting vote given by the chairman at a former meeting was illegal, he not having been formally elected.

A meeting will be held at the Mansion House on the 1st prox. in furtherance of a scheme for establishing a Pasteur institute in London. The Lord Mayor, who recently visited the institute at Paris, will probably preside.

The Samaritan Free Hospital for Women and Children is obliged to vacate the premises in Lower Seymour-street, Portman-square, which it has occupied since 1847, and has obtained a suitable piece of ground for its new premises in Marylebone-road.

Princess Christian has expressed her intention of opening the new buildings of the Cripples' Home and Industrial School for Girls, Northumberland House, 17a, Marylebone-road, on Saturday afternoon, June 29th, instead of July 1, as previously announced.

In response to the appeal on behalf of their "Special Disaster Fund," the Shipwrecked Fishermen and Mariners' Royal Benevolent Society has received contributions from the following City companies: The Goldsmiths', £100; the Mercers', £31. 10s.; and the Skinners', £10. 10s.

The Heathcote Hospital, erected by the combined authorities of the Mid-Warwickshire Sanitary District, for the reception and isolation of cases of infectious disease, was publicly opened by Alderman S. T. Wackrill, J.P., chairman of the Joint Hospital Board, on May 29th.

Mrs. Henry Fawcett and her daughter have jointly paid over to the National Association for Supplying Female Medical Aid to the Women of India the sum of £400 to be devoted towards the founding of two Scholarships, one in Calcutta, and the other in Bombay, for native female medical students.

A meeting of the Council of the Animals Institute was held last Saturday, to take into consideration a new ambulance carriage for the conveyance of injured horses, suggested by the Hon. Miss Hamilton Russell, and to arrange a series of lectures on "First Aid Ambulance in Street Accidents," by Professor Atkinson, F.R.C.V.S.

The death is announced of Professor David Boyes Smith, Professor of Military Medicine in the Army Medical School at Netley, which took place on the 3rd instant. This distinguished medical officer was appointed less than three years ago, in succession to Professor Maclean. His death is believed to be due to a tumour of the brain.

Among the additional donors to the funds of the Halifax New Infirmary is Mr. L. Clayton, who enters the £500 list. It is intimated, however, that if the £1,000 list can be brought up to 20, that he, and probably others now in the £500 list, will join the £1,000 donors. It will be most creditable to the town if it can display a list of 20 donors of £1,000 each towards this most necessary institution.

